# Exploring the Potential of Saphenous Vein Grafts Ex Vivo: A Model for Intimal Hyperplasia and Re-Endothelialization

**DOI:** 10.3390/jcm13164774

**Published:** 2024-08-14

**Authors:** Nur A’tiqah Haron, Mohamad Fikeri Ishak, Muhammad Dain Yazid, Ubashini Vijakumaran, Roszita Ibrahim, Raja Zahratul Azma Raja Sabudin, Hafiza Alauddin, Nur Ayub Md Ali, Hairulfaizi Haron, Muhammad Ishamuddin Ismail, Mohd Ramzisham Abdul Rahman, Nadiah Sulaiman

**Affiliations:** 1 Centre for Tissue Engineering and Regenerative Medicine, Faculty of Medicine, Universiti Kebangsaan Malaysia, Cheras, Kuala Lumpur 56000, Malaysiaubashinivijakumaran@ukm.edu.my (U.V.); 2 Department of Community Health, Faculty of Medicine, Universiti Kebangsaan Malaysia, Cheras, Kuala Lumpur 56000, Malaysia; 3 Department of Pathology, Faculty of Medicine, Universiti Kebangsaan Malaysia, Cheras, Kuala Lumpur 56000, Malaysia; 4 Department of Diagnostic Laboratory Services, Hospital Canselor Tuanku Muhriz, Universiti Kebangsaan Malaysia, Cheras, Kuala Lumpur 56000, Malaysia; 5 Department of Surgery, Faculty of Medicine, Universiti Kebangsaan Malaysia, Cheras, Kuala Lumpur 56000, Malaysia; 6 Heart and Lung Centre, Hospital Canselor Tuanku Muhriz, Universiti Kebangsaan Malaysia, Cheras, Kuala Lumpur 56000, Malaysia

**Keywords:** coronary artery bypass grafting, coronary artery disease, intimal hyperplasia, re-endothelialization, saphenous vein graft, intimal hyperplasia

## Abstract

Coronary artery bypass grafting (CABG) utilizing saphenous vein grafts (SVGs) stands as a fundamental approach to surgically treating coronary artery disease. However, the long-term success of CABG is often compromised by the development of intimal hyperplasia (IH) and subsequent graft failure. Understanding the mechanisms underlying this pathophysiology is crucial for improving graft patency and patient outcomes. **Objectives**: This study aims to explore the potential of an ex vivo model utilizing SVG to investigate IH and re-endothelialization. **Methods**: A thorough histological examination of 15 surplus SVG procured from CABG procedures at Hospital Canselor Tuanku Muhriz, Malaysia, was conducted to establish their baseline characteristics. **Results**: SVGs exhibited a mean diameter of 2.65 ± 0.93 mm with pre-existing IH averaging 0.42 ± 0.13 mm in thickness, alongside an observable lack of luminal endothelial cell lining. Analysis of extracellular matrix components, including collagen, elastin, and glycosaminoglycans, at baseline and after 7 days of ex vivo culture revealed no significant changes in collagen but demonstrated increased percentages of elastin and glycosaminoglycans. Despite unsuccessful attempts at re-endothelialization with blood outgrowth endothelial cells, the established ex vivo SVG IH model underscores the multifaceted nature of graft functionality and patency, characterized by IH presence, endothelial impairment, and extracellular matrix alterations post-CABG. **Conclusions**: The optimized ex vivo IH model provides a valuable platform for delving into the underlying mechanisms of IH formation and re-endothelialization of SVG. Further refinements are warranted, yet this model holds promise for future research aimed at enhancing graft durability and outcomes for CAD patients undergoing CABG.

## 1. Introduction

Saphenous vein grafts (SVGs) have been extensively employed as conduits in coronary artery bypass graft (CABG) procedures due to their availability and ease of harvesting [1,2]. However, long-term graft patency remains a significant challenge, with intimal hyperplasia being a major contributor to graft failure [3,4]. Intimal hyperplasia, characterized by excessive proliferation and migration of smooth muscle cells (SMCs) and extracellular matrix (ECM) deposition within the vessel wall, leads to neointimal thickening and luminal narrowing [5]. The development of intimal hyperplasia in SVG is a multifactorial process influenced by various cellular and molecular mechanisms [6]. Endothelial dysfunction serves as a precursor to intimal hyperplasia, a process central to vascular pathophysiology. Endothelial dysfunction in vein grafts typically arises from mechanical injury during harvesting, surgical manipulation, and exposure to arterial hemodynamics, resulting in endothelial denudation and dysfunction. Thus, re-endothelialization is a crucial process for maintaining graft patency and preventing intimal hyperplasia.

Endothelial cells (ECs) are critical in vein graft disease (VGD), especially in the formation of intimal hyperplasia. Normal ECs create a protective layer, separating blood from the vessel wall. If this layer is compromised, it can trigger serious pathological consequences [7]. Healthy ECs are able to produce nitric oxide (NO), which helps regulate blood vessel tension and prevents excessive growth of SMC. A key indicator of endothelial dysfunction is the reduction in nitric oxide (NO) activity. This decrease in NO activity is closely linked to the advancement of VGD [8,9]. The re-establishment of a functional endothelial monolayer regulates SMC proliferation, inhibits platelet activation, and maintains vascular homeostasis [10].

Proliferation and migration of SMCs to the lumen, initiated by injury to the endothelial layer, might be due to either a pre-existing condition caused by risk factors or previous intervention [11]. Vascular smooth muscle cells (SMCs) play a crucial role in the development of various vascular lesions, including intimal hyperplasia (IH) in vein grafts [5]. Research has consistently shown that mature SMCs are the primary cellular component in IH, regardless of the extent of neointima formation. The contribution of SMCs to IH involves several interconnected mechanisms [12,13,14,15]. Upon vascular injury or stress, SMCs undergo phenotypic switching from a contractile to a synthetic state [12], enabling them to proliferate and migrate [13]. These activated SMCs then increase cell division, leading to intimal layer thickening, and migrate from the media to the intima, accumulating in the vessel wall’s inner layer. In their synthetic state, SMCs also produce excessive extracellular matrix components, further contributing to intimal thickening [14]. Additionally, SMCs participate in inflammatory responses by producing and responding to various mediators, perpetuating vascular remodeling. They also interact with endothelial and inflammatory cells, influencing the overall progression of IH [15,16]. Collectively, these processes result in significant thickening of the intimal layer, which narrows the vessel lumen and potentially compromises blood flow in the vein graft.

Understanding the intricate interplay between intimal hyperplasia and re-endothelialization is essential for improving the long-term outcomes of SVG in CABG surgery. Developing a suitable experimental model is crucial to investigating these processes. An ex vivo model offers an advantageous approach to studying the mechanisms underlying intimal hyperplasia and re-endothelialization in a controlled environment. In recent years, ex vivo models utilizing saphenous vein grafts have gained attention in cardiovascular research. These models involve the removal of SVG during CABG surgery, followed by their subsequent culture and manipulation under controlled laboratory conditions [17,18,19,20,21,22]. The ex vivo environment allows for investigating cellular and molecular events associated with intimal hyperplasia and re-endothelialization, providing insights into the underlying mechanisms and potential therapeutic strategies.

The ex vivo SVG model offers several advantages in studying intimal hyperplasia and re-endothelialization. First, the use of human grafts enables the investigation of disease-specific factors and patient-specific responses, enhancing the clinical relevance of the findings [23]. Second, the ex vivo setting allows for the precise control of experimental conditions, such as oxygen tension, nutrient supply, and pharmacological interventions [17,24]. This control enables the evaluation of specific cellular responses and the effects of various interventions on intimal hyperplasia and re-endothelialization. Various approaches can be employed to enhance re-endothelialization and mitigate intimal hyperplasia. Strategies may include applying growth factors, the introduction of biomolecules, or gene therapy techniques [25,26]. Potential therapeutic targets and approaches can be identified by evaluating the effects of these interventions on cellular behavior and graft remodeling.

Manipulation of the ex vivo model allows for the investigation of factors influencing intimal hyperplasia and re-endothelialization. Therefore, the initial characterization and further use of surplus saphenous vein grafts obtained during CABG surgery in establishing valuable ex vivo models are significant, hence the suitable aim of this study.

## 2. Materials and Methods

### 2.1. Subjects

Tissue samples: Patients managed by cardiothoracic surgeons in the Department of Surgery, Hospital Canselor Tuanku Muhriz, Faculty of Medicine, Universiti Kebangsaan Malaysia were identified, and consent was obtained prior to the CABG procedure in accordance with guidelines set by the Ethics and Research Committee, Faculty of Medicine, UKM (UKM PPI/111/8/JEP-2020-540). Patient data were collected from the CASEMIX MY-DRG^®^ database. The CASEMIX MY-DRG^®^ database is a classification method for patients based on their treatment history field utilized by HCTM [27].

Blood samples: All experiments involving human blood adhered to the approved guidelines set by the Ethics and Research Committee, Faculty of Medicine, UKM (UKM PPI/111/8/JEP-2020-540) and were conducted only after obtaining informed, signed consent from 14 healthy human donors, with an average age of 31 (ranging from 23 to 41), and 57% of the donors were female.

### 2.2. Sample Collection

#### 2.2.1. Tissue Samples

A total of 35 cases of CABG surgery were performed in HCTM, and surplus of saphenous vein samples were collected at the end of surgery. Only 15 samples (*n* = 15) out of 35 of SVG were processed and analyzed. All vein collection was performed by the surgeons involved through a longitudinal open incision where the perivascular tissue was stripped, side branches ligated, and veins were then cannulated with minimal manipulation to avoid endothelial damage. Harvested veins were kept in a buffered saline bath before implantation, and sections of the left-over grafts that were injured internally by forceps or clamps were removed after a thorough examination of the segments. Surplus sections, free of injury, were used for further histological analysis. The samples were placed in a sterile bottle filled with buffered saline and transported to the laboratory.

Positive control: Umbilical cord samples, approximately 10–15 cm in length, were obtained from consenting healthy donors undergoing caesarean sections at Hospital Canselor Tuanku Mukhriz in Kuala Lumpur, Malaysia. Patients were informed, and their consent was secured before the scheduled surgeries. The umbilical arteries were carefully extracted from these cord samples under sterile conditions. To ensure the integrity of the samples, the time between delivery and processing was strictly monitored and kept under 24 h. Once isolated, the arteries were cleansed using Dulbecco’s phosphate-buffered saline (DPBS, Gibco) and a 1% antibiotic-antimycotic solution (AA, Corning) in preparation for the denudation protocol. This cleaning step was crucial to maintaining the quality and sterility of the samples for subsequent experimental procedures.

#### 2.2.2. Blood Samples

Twenty milliliters of peripheral blood was collected from healthy donors. Venipuncture was used to collect the blood drawn into Vacutainer lithium heparin tubes (BD Bioscience, San Jose, CA, USA). All blood was processed within 4 h of collection to maintain sample integrity.

### 2.3. Cell and Tissue Culture

#### 2.3.1. Tissue Culture

Intimal hyperplasia model: Surplus saphenous vein samples were longitudinally cut to expose the lumen and further sectioned into ≈1 cm × 1 cm in size. According to sample availability, tissue sections were divided into two groups, i.e., direct method (DM) and scrapping method (SM), and each group was further prepared for 4 different culture days (days 1, 3, 5, and 7). In the DM group, tissue sections with four corners were fixed onto a Sylgard-containing glass Petri dish with minute pins. Prolonged tissue culture was achieved with a change of 2 mL of endothelial cell growth medium MV (ECGM-MV, C-22120, Promocell, Heidelberg, Germany) every other day. Tissue section lumens for the SM group were first scraped to ensure no endothelial cells remained and were then treated the same as the DM group. Tissue sections were fixed at the end of each culturing condition for further histological analysis.

Re-endothelialization model: Surplus saphenous vein samples were prepared as explained in the SM intimal hyperplasia model set-up. The tissue sections were divided into two groups, i.e., control (no cell seeded) and treatment group, which was seeded with blood outgrowth endothelial cells (BOECs) at a density of 10,000 cells/cm^2^. Each group was further prepared for prolonged tissue culture at 4 different culture days (days 1, 3, 5, and 7) with ECGM-MV media changed every other day. Tissue sections were fixed at the end of each culturing condition for further histological analysis.

Positive control sample: To serve as a positive control, umbilical artery (UA) samples were carefully extracted from the Wharton’s jelly (WJ) under sterile conditions. This extraction process involved the use of forceps and a scalpel to make an incision in the umbilical cord. Before the isolation procedure, the sample was thoroughly rinsed with Dulbecco’s phosphate-buffered saline (DPBS) to eliminate any blood residue. Following the cleaning, the isolated UA was cut into segments of approximately 1 cm in length. These segments were then immediately preserved by immersion in a 10% neutral buffered formalin (NBF) solution for fixation. This preparation method ensured the UA samples were properly preserved for use as reliable positive controls in subsequent analyses.

#### 2.3.2. Cell Culture

Blood samples: Lymphoprep was added to a SepMate tube at room temperature per the manufacturer’s instructions. The whole blood was mixed with an equal volume of Dulbecco’s phosphate-buffered saline (DPBS) at room temperature and gently added to the tube. The tube was then centrifuged at 1200× *g* for 10 min at 21 °C with acceleration and deceleration. After centrifugation, the resulting plasma and peripheral blood mononuclear cells (PBMC) were carefully transferred into a separate 50 mL tube and washed with Dulbecco’s Modified Eagle Medium (DMEM)-fetal bovine serum (FBS) at 500× *g* for 10 min at 4 °C. Following the washing step, PBMCs were resuspended in DMEM-FBS. The enumeration and viability of PBMCs were assessed using trypan blue and a hemocytometer. The cells were then cultured on a fibronectin-coated 6-well plate with ECGM-MV media changed every other day until confluency to obtain a sustainable BOEC population. The cells underwent further expansion and were pooled to achieve enough numbers when used in the re-endothelialization model.

### 2.4. Histological Analysis

Sampled tissues were fixed in 10% neutral buffered formalin (NBF) for 24 h prior to further histological analysis. Fixed tissue samples were placed in histology processing cassettes and sent to the Department of Diagnostic Laboratory Services to be processed in the Excelsior Tissue Processor (Epredia, Kalamazoo, MI, USA) before paraffin embedding. All paraffin-embedded samples were stored at room temperature before further analysis. A Leica 2255 semi-automated rotary microtome was used to cut 3 to 5 μm of the paraffin-embedded samples, and they were adhered onto Superfrost Plus slides (4951PLUS; Thermo Fisher Scientific, Princeton, NJ, USA) for histological staining. Tissue slices on slides were deparaffinized and rehydrated. Rehydrated slides were stained with H&E as previously described [28]. Additionally, slides for Picro-Sirius Red (ab245887, Abcam, Fremont, CA, USA), Elastin Van Gieson (ab150667, Abcam, Fremont, CA, USA), and Alcian Blue (ab150662, Abcam, Fremont, CA, USA) staining were stained following the manufacturer’s recommended protocols. Stained slides were dehydrated before being mounted with a quick-hardening mounting medium (80610, Cancer Diagnostic, Colleyville Center, TX, USA) before coverslip placement and allowed to dry. Observation and image acquisition were performed at 20× or 40× (oil immersion) magnification. Area of interest quantification was performed using publicly accessible image processing software, Fiji 1.53o.

### 2.5. Immunofluorescence (IF)

Tissue sections on slides were warmed on a slide warmer for around 24 h to ensure that the tissue sample did not detach from the slide during antigen retrieval. After 24 h, the slides were placed in a pressure cooker at 110 °C for 60 to 90 min with a pH 6 citrate antigen retrieval buffer. The slides were placed in a Sequenza staining device and washed with phosphate-buffered saline, PBS (2235032, Gibco Thermo Fisher Scientific, New Jersey, USA). The tissue sections were then blocked with 10% goat serum (G9023, Sigma Life Science, Burlington, MA, USA) and incubated for 1 h at room temperature. Primary antibodies include CD31-1:500 (NB600-1475, Novus Biologicals, Centennial, CO, USA), αSMA-1:100 (ab7817, Abcam, Waltham, CA, USA), CD309-1:100 (NBP2-36428, Novus Biologicals, Centennial, CO, USA), VEGFR-1:100 (NB120-16518, Novus Biologicals, Colorado, USA), CD146-1:100 (NBP2-44512, Novus Biologicals, Centennial, CO, USA), CD133-1:250, (NB120-16518, Novus Biologicals, Centennial, CO, USA), and CD45-1:100 (NB100-77417, Novus Biologicals, Centennial, CO, USA). Secondary antibodies include goat anti-mouse 488-1:100 (ab150077, Abcam, Milpitas, CA, USA), and goat anti-mouse 594-1:100 (ab150080, Abcam, Milpitas, CA, USA).

The primary antibody was pipetted directly onto tissue sections and incubated overnight at 4–6 °C. The slides were washed with PBS before adding the secondary antibody on the following day and incubated for 2 h at room temperature. Counterstaining of the nuclei, with 4′,6-diamidino-2-phenylindole, DAPI (1733809, Thermo Fisher Scientific, Branchburg, NJ, USA) was performed with a 40 min incubation. The stained slides were mounted with a water-based mounting solution before observation and image acquisition.

Positive control: The tissue sections underwent antigen retrieval using a pressure-activated, high-temperature method in a pH 6 citrate buffer solution (1X antigen retrieval solution, Dako). Following this, the sections were treated with a blocking solution of 10% goat serum (Sigma-Aldrich, St. Louis, MO, USA) in PBS for one hour. The samples were then incubated overnight at 4 °C with primary antibodies: anti-CD31 and anti-alpha smooth muscle actin (both diluted 1:200, Abcam) in a 1% goat serum PBS solution. After three wash cycles, the sections were exposed to AlexaFluor-conjugated secondary antibodies (diluted 1:500, Abcam) in 1% goat serum for one hour at 37 °C. Nuclear counterstaining was performed using DAPI (diluted 1:15000 in PBS, Sigma-Aldrich, USA) for 40 min at room temperature. The prepared slides were sealed using CoverSeal aqueous mounting solution (Cancer Diagnostics, Colleyville Center, Colleyville, TX, USA) prior to observation and image processing.

### 2.6. Image Processing

All histological staining, i.e., hematoxylin–eosin, picrosirius red, elastin Van Gieson, and Alcian blue stained slides, was imaged by an Olympus BX40 microscope attached to a DP275-megapixel camera to acquire high definition (HD) images. Image processing software Fiji was used to quantify all the color pixels of the stained tissue [29]. The circumference determined the vein lumen diameter. The diameter is derived from the circumference measurement of H&E-stained images using the equation stated below:

Diameter, D = Circumference/π

As for intima medial thickness, a measurement was taken from tunica intima to tunica media. An average thickness from 5 areas of each image was measured and analyzed. Intimal hyperplasia thickness was measured between the intima and the start of the concentrically aligned smooth muscle cells. The presence of intimal hyperplasia in each sample was not uniformly located around the whole lumen.

The percentage of endothelial coverage in the lumen was obtained via immunofluorescent staining of tissue sections using the endothelial-specific marker CD31 using the specified formula below:

Percentage of endothelial coverage = (Total Endothelial Coverage on Lumen)/(Lumen Perimeter) × 100%

### 2.7. Statistical Analysis

All analyses were performed with at least four biological replicates. Statistical analyses were performed using a mixed-effects model with Geisser–Greenhouse correction and Tukey’s multiple comparisons test due to missing values in identifying the significant differences between the different test groups and culturing days. Both tests were performed via GraphPad Prism version 9 (GraphPad Software). The data were collected and presented as the mean ± standard deviation and *p* = 0.05.

## 3. Results

### 3.1. Demographic, Clinical Characteristics and Outcome

**Figure 1:**
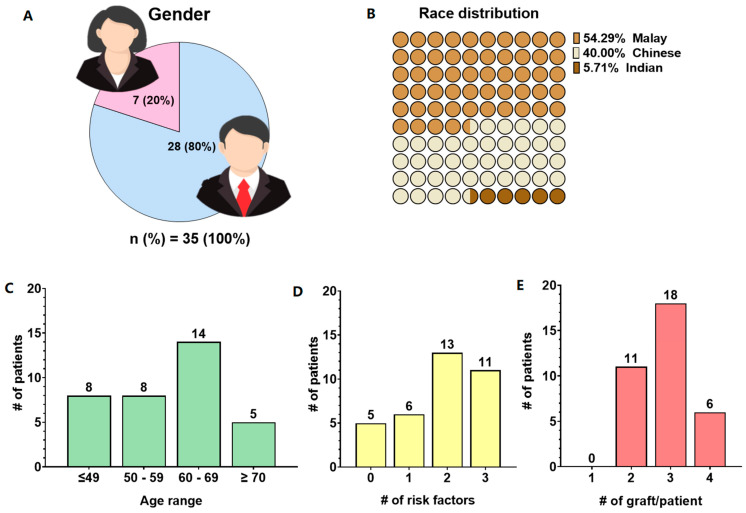
Patients’ demographic and clinical characteristics. (**A**) Gender distribution, (**B**) race distribution, (**C**) patients’ age group distribution, (**D**) number of CVD associated risk factors per patient, and (**E**) number of grafts needed per patient. Number of subjects, *n* = 35.

A total of 35 patients underwent CABG procedures between September 2020 and December 2021. Patients’ demographic data are tabulated in Table 1. Patients’ demographic and clinical characteristics were laid out as in Figure 1. A higher proportion of patients undergoing coronary artery bypass graft (CABG) at HCTM are male (80%: 28 males, 20%: 7 females) (Figure 1A). In terms of the racial distribution of CABG patients at HCTM, which reflects the Malaysian population, with the majority being Malay (54.29%), followed by Chinese (40%), and the least represented group being of Indian (5.71%) racial background (Figure 1B). The mean age of patients requiring CABG procedures at HCTM is 59 ± 9.2 years, with a higher prevalence in the 60- to 69-year-old age group (Figure 1C). Most patients (60%) were presented with more than two cardiovascular disease (CVD) risk factors, as shown in Figure 1D, including hypertension (80%), diabetes mellitus type II (37%), hyperlipidemia (63%), and obesity (6%). The past medical history of this patient cohort includes chronic kidney disease (11%), fatty liver disease (6%), and transient ischemic attack (TIA) or mini stroke (9%), as tabulated in Table 1. All patients received statin therapy prior to surgery, and none had pre-existing lower limb venous insufficiency. The majority of this patient’s cohort has a normal left ventricular ejection fraction (LVEF). More than 68% of patients required more than 2 graph replacements, signifying multivessel disease in most of the cohort (Figure 1E).

### 3.2. Variability in Lumen Diameter, Medial Thickness, and Intimal Hyperplasia

**Figure 2:**
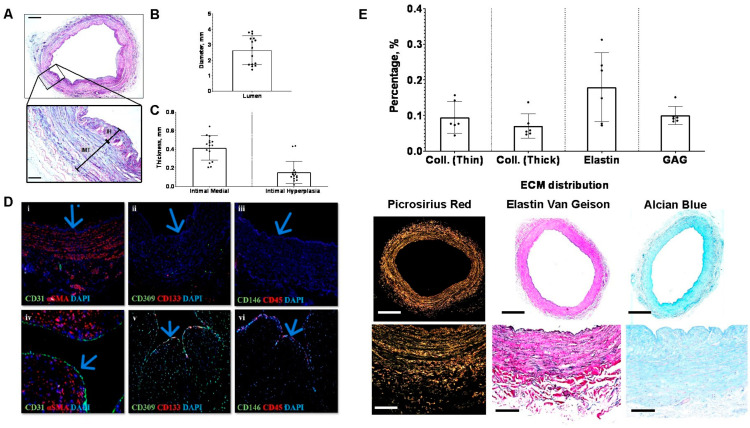
Vein graft histological analysis. (**A**) Representative image of intimal hyperplasia. Arrows denote the medial thickness (MT) and intimal hyperplasia (IH) at 200× magnification. (**B**) Measurement of vessel diameter. (**C**) Measurement of intimal medial and intimal hyperplasia thickness were performed using Fiji software. (**D**) The sections of the saphenous vein (top panel) and umbilical cord (bottom panel) as a positive control were stained with (**i**,**iv**) CD31 + αSMA, (**ii**,**v**) CD309 + CD133, and (**iii**,**vi**) CD146 + CD45. Blue arrow shows the endothelial layer of SV and UA, respectively. (**E**) Percentage of extracellular matrix (%) using special staining to visualize thin and thick collagen, elastin, and glycosaminoglycans. Scale bar represents 200 µm and 2 mm for 40× and 200× magnification, respectively. All analysis was performed with *n* = 6.

Fifteen saphenous veins were collected for histological analysis after undergoing additional screening by surgeons and considering the availability of surplus tissue. Figure 2A and the inset depict a representative image of an H&E-stained vein. The data collectively demonstrate that the vein diameter ranges from 1.4 mm to 3.88 mm, with a mean of 2.65 ± 0.93 mm (Figure 2B). Intima medial thickness, encompassing the tunica intima and tunica media of the vein wall, ranges from 0.208 mm to 0.647 mm, with an average of 0.42 ± 0.13 mm. The distribution of intimal hyperplasia within the vein sections is not uniform around the lumen. On average, the thickness of intimal hyperplasia is 0.15 ± 0.12 mm (Figure 2C).

### 3.3. Surplus Saphenous Vein Grafts Endothelial Cells Coverage

An umbilical artery was stained as a positive control tissue where CD31, CD309 (alternatively known as vascular endothelial growth factor receptor, VEGFR), CD133 (a transmembrane glycoprotein also known as prominin-1), CD146 (also known as cell surface glycoprotein MUC18), and CD45 (a leucocyte marker) are expressed. CD31, a vascular endothelial marker, confirms the presence of endothelial cells in the lumen of the umbilical artery (Figure 2D(iv)), where none were detected in the vein graft (Figure 2D(i)). CD309 and CD133, as well as CD146 and CD45, were also apparent in the umbilical artery (Figure 2D(v,vi)) but not in the vein graft (Figure 2D(ii,iii)), respectively.

### 3.4. Baseline Saphenous Vein Grafts Extracellular Matrix Composition

Thin (greenish yellow) and thick collagen (reddish orange) were observed under a polarized light microscope with Picrosirius red staining (PSR). The collagen content, i.e., thick and thin, in the surplus saphenous vein collected is approximately 0.1 ± 0.04% and 0.07 ± 0.03%, respectively. Vein stained with elastin Van Gieson enables the visualization of elastic fibers stained dark purple. The percentage of elastin varied in each sample, as two samples had less than 0.1% of elastin with a mean of 0.2 ± 0.1%. glycosaminoglycans (GAGs) in veins are stained blue with Alcian blue (AB) staining. The percentage of GAG in five samples is found to be lower than 0.1%, while only one sample has 0.15% of GAG with an average of 0.1 ± 0.03% (Figure 2E).

### 3.5. Surplus Saphenous Vein Grafts Intimal Hyperplasia Ex Vivo Model (DM vs. SM)

**Figure 3:**
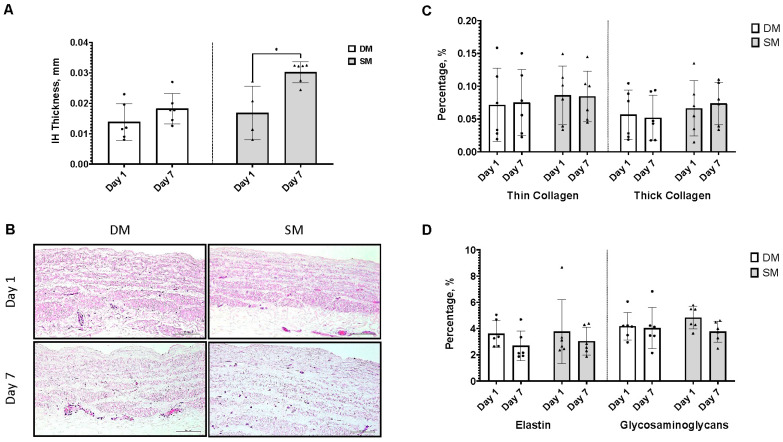
Analysis of intimal hyperplasia of surplus saphenous vein grafts ex vivo model. (**A**) Intimal hyperplasia (IH) thickness on day 1 and day 7 for direct method (DM) and scrapping method (SM). The triangle shows there is significant difference observed for group SM, * *p* < 0.05. (**B**) Representative image of IH thickness for both DM and SM on day 1 and day 7. (**C**) Percentage of thin and thick collagen in day 1 and day 7 of DM and SM. (**D**) Percentage of elastin and glycosaminoglycans on day 1 and day 7 of DM and SM. Quantification was performed by using Fiji software. The scale bar represents 100 µm. Analysis was performed with *n* = 6. • representing each data point or replicates.

In establishing an ex vivo intimal hyperplasia model, the scrapping method (SM) supersedes the direct method (DM), as at day 7 of culture, the IH thickness was significantly higher in SM (0.03 ± 0.003 mm) than in DM (0.018 ± 0.005 mm) (Figure 3A). No differences were observed in the ECM of both ex vivo models (Figure 3C,D).

### 3.6. Re-Endothelialization of Intimal Hyperplasia Ex Vivo Model (SM vs. BOEC)

**Figure 4:**
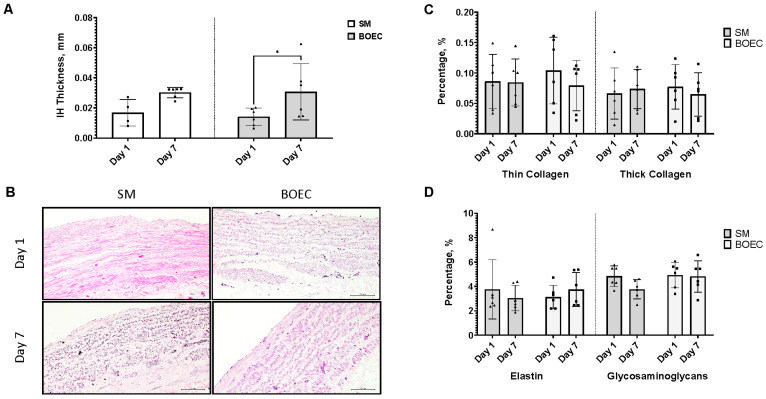
Re-endothelialization of saphenous vein grafts intimal hyperplasia ex vivo model. (**A**) Intimal hyperplasia (IH) thickness on day 1 and day 7 for scrapping method (SM) and blood outgrowth endothelial cell (BOEC). The triangle shows there is a significant difference was observed for group BOEC, * *p* < 0.05. (**B**) Representative image of IH thickness for both SM and BOEC on day 1 and day 7. (**C**) Percentage of thin and thick collagen on day 1 and day 7 for group SM and BOEC. (**D**) Percentage of elastin and glycosaminoglycans on day 1 and day 7 of SM and BOEC groups. Quantification was performed using Fiji software. The scale bar represents 100 µm. Analysis was performed with *n* = 6. • representing each data point or replicates.

Re-endothelialization of the ex vivo intimal hyperplasia model with BOEC shows no significant difference in IH thickness between the control (SM model) and the seeded SM model (BOEC) at day 7 of culture. Although 50% of the BOEC seeded group IH thickness is observed to be lower than the control, the overall means of both groups are 0.03 ± 0.02 mm (BOEC) and 0.03 ± 0.003 mm (SM), respectively (Figure 4A). No differences were observed in the ECM of both control and seeded IH ex vivo models (Figure 4C,D).

## 4. Discussion

The demographic data collected show that ≥60% of patients who underwent the CABG procedure in HCTM were older than 60 years, with ≥2 CVD risk factors that required ≥3 grafts for CABG, echoing previous national findings that spark a national health urgency to combat CVD and its associated risk factors [30,31]. The sampled population suffers mainly from hypertension, which has shown a strong association with an increase in common carotid artery (CCA) intimal media thickness (IMT) in several independent studies [32,33]. Although the cited studies conducted ultrasound analyses of CCA, the correlation between hypertension and IMT should be noted. It is a credible predictor of vein graft long-term patency, especially in this patient cohort, where 71% had hypertension. The presence of neointimal hyperplasia at baseline is an exclusive phenomenon that occurs in vein grafts, threatening the long-term patency of the graft post-CABG [34].

Four samples showed no EC, and two samples reported a low percentage of endothelial coverage (EC) of the vein lumen. This may be attributed to the sample harvesting and processing technique, where conventional open saphenous vein harvesting (OSVH) was practiced [35]. The conventional method for harvesting the long saphenous vein (LSV) involves an open approach, requiring a lengthy incision from groin to ankle along the medial aspect, with care taken to minimize vein manipulation [36,37,38]. The preparation process for saphenous vein grafts (SVGs) includes several steps such as exposure, dissection, distension of the lumen, ligation of side branches, and preservation in a physiological solution. However, this process inevitably results in significant endothelial damage [39]. This traditional technique carries risks of considerable wound complications for certain patients, such as post-surgery pain, infections, and slow wound healing, potentially extending hospital stays and necessitating ongoing wound care in the community [40]. While surgical skill is the primary determinant of wound outcomes, the closure method also plays a role. In recent years, endoscopic vein harvesting (EVH) has become increasingly popular to mitigate the pain and infection risks associated with the open procedure [37]. However, concerns persist not only about long-term graft patency but also about complications at the harvest site itself [41]. These potential issues include chronic postoperative pain or abnormal sensations, wound infections, the development of varicose veins in the lower extremities, and leg swelling due to reduced venous drainage following vein removal [41].

Linking the clinical to histological data, in terms of ECM, higher levels of thin collagen were observed than thick collagen, previously revealed to co-localize with type I collagen in the collagen fibril. Thin and thick collagen are associated with collagen type III and type I, respectively, and both are deemed crucial for fibrillogenesis to maintain the mechanical stability of blood vessels [42]. Further histological analysis of vein grafts from CABG patients shows evidence of pre-existing intimal hyperplasia and displays low to non-existent endothelial coverage. Endothelial disruption causes smooth muscle cell (SMC) migration, where the abnormal growth of SMCs is crucial in the pathogenesis and progression of intimal hyperplasia [11]. A major limitation of aortocoronary and peripheral vascular bypass surgeries is the failure of SVG bypass conduits due to intimal hyperplasia [4,43]. Intimal hyperplasia was observed in most of the veins collected, although they were not homogeneously distributed around the lumen. Damage to the endothelium layer also contributes to protein deposition in the ECM, which encourages the initiation of growth and migration of SMCs [11]. The phenotypic change of SMCs from contractile to synthetic occurs during the pathophysiology of IH. This transition is associated with the increased production of ECM components such as collagen [44]. Platelet-derived extracellular vesicles were shown to stimulate SMCs to increase collagen synthesis in the carotid artery injury model [45]. Therefore, collagen deposition provides clues on IH progression. The differences in thin and thick collagen composition in the vein graft collected may be used as a baseline for vein graft quality and might be an indicator of tissue remodeling or damage. As demonstrated in tumor progression, higher greenish-yellow than yellowish-orange indicates higher immature or loosely packed collagen than more structurally intact mature collagen [46]. Collagen and elastin are uniformly organized from intima to adventitia; this organization is vital in outward vascular remodeling [47]. Elastic fibers and laminae, on the other hand, inhibit SMC proliferation and deter the formation of intimal hyperplasia [48]. Elastin organization is apparent in arteries but not in veins, which may be one of the contributing factors to low long-term vein graft patency. Unfortunately, the synthetic SMC phenotype further degrades elastin and deposits cross-linked collagens, causing stiff vascular walls [49].

On the other hand, functional endothelial cells have the potential to inhibit IH by suppressing the adhesion of platelets, thrombus formation, and initial migration of SMCs [50]. Endothelium protection and functionality are determined by activating the glycosaminoglycans (GAG) layer, which works as a buffer and force transmitter under favorable vascular conditions [51]. GAG is especially important on the luminal surface of blood vessels as it provides a suitable bed for endothelial cells to anchor on. Histological staining of GAG via Alcian blue staining is non-specific; thus, analyzing the integrity of endothelial cells through immunohistochemical staining of specific markers is imperative. Platelet endothelial cell adhesion molecule-1 (PECAM-1 or CD31) is a common endothelial marker used to characterize endothelial cells in any tissue [52]. The present study revealed deficient levels of the endothelial marker CD31.

As previously reported, transplanted endothelial progenitor cells (EPCs) strongly boost re-endothelialization and inhibit neointimal hyperplasia [53]. Hence, in this study, SVGs were stained with CD309, CD146, and CD133, markers of EPCs, to observe any potential EPC recruitment on the vein graft, especially in the area with pre-existing IH after SVG extraction [54]. EPCs are highly proliferative and can differentiate into mature endothelial cells that are functional, thus repairing the endothelial layer on the injured vascular bed [55]. Endothelial cells isolated from saphenous veins were reported to possess comparable “stemness” surface marker expression, proliferation, and vasculogenic ability as cord blood-derived endothelial cells [56]. Unfortunately, a lack of CD309 and CD146 staining was observed on SVG, showing no repair mechanism was in place on extracted tissue, especially those with pre-existing IH. Interestingly, CD133 expression in the tunica media of preoperative saphenous veins has strongly predicted early vein graft failure, which was not observed in this study [57]. CD133 expression in the media indicates initial tissue remodeling has gone awry, leading to inward remodeling of the graft, causing further intimal hyperplasia progression [58,59]. An umbilical cord was used as a positive tissue control for all endothelial markers to validate the markers, as we could not acquire a healthy saphenous vein or artery.

Our study aligns with the previous report of pre-existing IH observed in SVG harvested for CABG surgery. Correspondingly, pre-existing IH was apparent in the cephalic vein of kidney failure patients during arteriovenous fistula construction, too [60]. A loss of endothelial layer and internal lamina (30%) was also observed with intimal hyperplasia [60]. Similarly, a cohort of 96 AVF surgery patients from a single center reported that 98% of the patients had pre-existing IH (>0.05 mm), with 10% of AVF failing to mature [6]. This evidence supports our findings of pre-existing IH in the native saphenous vein. Hence, our data collection of pre-existing IH could drive more research into preserving the integrity of vein grafts and slowing further development of IH in vein grafts. The present study collates and correlates the demographic data and histological analysis of vein grafts used in a single center, i.e., HCTM. These preliminary reports were done to demonstrate the prevalence of pre-existing intimal hyperplasia in patients undergoing CABG at HCTM and the associated risk factors. The baseline ECM components, i.e., collagen, elastin, and GAGs, in vein grafts are analyzed and reported in this present study to establish comparable data for future studies in re-endothelialization of vascular grafts, which is a viable approach to increasing the long-term patency of vein grafts.

Drug therapy targeting distinct stages of neointimal hyperplasia pathogenesis, particularly to block SMC migration and proliferation, can prevent further progression and deterioration. Statins, for example, hinder the development and progression of intimal hyperplasia by inhibiting matrix metalloproteinase (MMP) activity in a vein organ culture model [61]. MMP is a known contributor to atherosclerosis development; unstable CAD patients had significantly higher MMP2 levels and activity than stable CAD patients and healthy individuals [62]. Clinically, lower mortality and MACE post-CABG were observed with statin treatment [63]. Statin therapy post-CABG is administered to this patient cohort to improve vein graft patency, leading to a lower percentage of MACE recorded.

In the context of saphenous vein graft failure in coronary artery bypass grafting (CABG) patients, several key signaling pathways are implicated in intimal hyperplasia and endothelial dysfunction. Intimal hyperplasia is primarily driven by inflammatory and growth factor signaling pathways. The NF-κB pathway, which regulates pro-inflammatory cytokine expression, is activated in response to endothelial injury, promoting inflammation and vascular smooth muscle cell proliferation [64]. Concurrently, the platelet-derived growth factor (PDGF) pathway stimulates smooth muscle cell migration and proliferation into the neointima, exacerbating graft failure [65]. Additionally, endothelial dysfunction in vein grafts is often associated with reduced nitric oxide (NO) production due to diminished endothelial nitric oxide synthase (eNOS) activity, leading to impaired vasodilation and increased oxidative stress [66]. The RhoA/Rho-kinase pathway also contributes by enhancing vascular tone and smooth muscle contraction, further compounding the issue [67]. Furthermore, oxidative stress, mediated by NADPH oxidase, generates reactive oxygen species (ROS) that damage endothelial cells and promote inflammation, intensifying intimal hyperplasia [68]. These signaling pathways collectively contribute to the pathological changes observed in saphenous vein grafts, highlighting the complex molecular mechanisms underlying graft failure and emphasizing the need for targeted therapeutic strategies to address these issues effectively [69]. We are actively working to address these issues by first establishing a robust model of intimal hyperplasia and endothelial dysfunction [70], hence the validation of models for future use of potential natural compounds that target these key pathways. Compounds with anti-inflammatory, antioxidant, and vasoprotective properties are hypothesized to mitigate the molecular mechanisms contributing to graft failure and improve outcomes for CABG patients [71].

Several limitations impacted this study, including the COVID-19 pandemic, which led to the imposition of a movement control order (MCO) by the Malaysian government shortly after the study’s funding was approved. This resulted in laboratory closures and the postponement of CABG surgeries for cardiovascular disease (CVD) patients, effectively halting our research.

Additionally, obtaining the required number of saphenous vein (SV) samples for the ex vivo vein graft culture model was delayed until after ethical approval in August 2020, during the conditional MCO (CMCO), which allows some but limited laboratory access. The pandemic also hampered efforts to optimize the samples, and variability in patient demographics, such as age and comorbidities, further influenced the results of the ex vivo model and the re-endothelialization process. The small sample size and the brief duration of the study added to these challenges.

Recruitment of healthy donors for blood outgrowth endothelial cell (BOEC) culture was also affected by the pandemic. BOECs, being a rare subpopulation of mononuclear cells with a frequency of 1 to 4 cells per mL of blood, made it difficult to obtain sufficient cells for cultivation. To address this, blood samples were pooled to achieve volumes of 10 to 15 mL, which was necessary for successful cell extraction and culture. Furthermore, endothelial cells require large numbers for effective in vitro culturing and maintenance of their phenotype, which we have successfully done as depicted in Supplementary Figure S1.

Despite these limitations, we believe that the findings of our study are significant and offer valuable insights into the challenges and potential solutions related to vein graft pathology. We are confident that these results will contribute meaningfully to the scientific community and stimulate further research in this area.

## 5. Conclusions

In conclusion, this study offers important insights into the histological features and behavior of SVGs used in CABG surgery. The observed presence of intimal hyperplasia, endothelial loss, and alterations in the ECM underscores the intricate nature of graft patency and functionality. Analyzing the grafts’ behavior and characteristics post-extraction and in an ex vivo setting provides valuable information on the SVG structure and the impact of prolonged culture. While further optimization is needed, the SVG ex vivo model is a promising foundation for future research to enhance graft patency and functionality, thereby offering improved prognoses for patients with CAD.

## Figures and Tables

**Table 1:** Patient’s demographic and clinical data.

Patient Characteristics	Patient (*n* = 35)
Mean age in years ± standard deviation	59 ± 9.2
CVD risk factors: Diabetes mellitus, type II, *n* (%)	13 (37%)
Hypertension, *n* (%)	28 (80%)
Hyperlipidemia, *n* (%)	22 (63%)
Obesity, *n* (%)	2 (6%)
History of smoking, *n* (%)	14 (40%)
Past medical history: Chronic kidney disease, *n* (%)	4 (11%)
Fatty liver disease, *n* (%)	2 (6%)
Transient ischemic attack (TIA), or mini stroke, *n* (%)	3 (9%)
LVEF%: Reduced (≤40%), *n* (%)	4 (11%)
Borderline (41–49%), *n* (%)	10 (29%)
Normal (50–70%), *n* (%)	21 (60%)

## Data Availability

The data supporting this study can be obtained by contacting the corresponding author. Access to the dataset requires approval from the Ethics and Research Committee, Faculty of Medicine and Hospital Canselor Tuanku Muhriz, Malaysia, to ensure compliance with patient confidentiality and ethical standards. Requests for data access should be directed to nadiahsulaiman@ukm.edu.my.

## Supplementary Materials

The following supporting information can be downloaded at: https://www.mdpi.com/article/10.3390/jcm13164774/s1, Figure S1: BOEC characterization.
