# Supplementary material for: Exploring the Potential of Saphenous Vein Grafts Ex Vivo: A Model for Intimal Hyperplasia and Re-Endothelialization
Source: J Clin Med. 2024 Aug 14;13(16):4774. doi: 10.3390/jcm13164774 (PMC11355503; doi:10.3390/jcm13164774)
Supplement: Supplementary file 1 [file jcm-13-04774-s001.zip › jcm-3092198-supplementary.pdf]

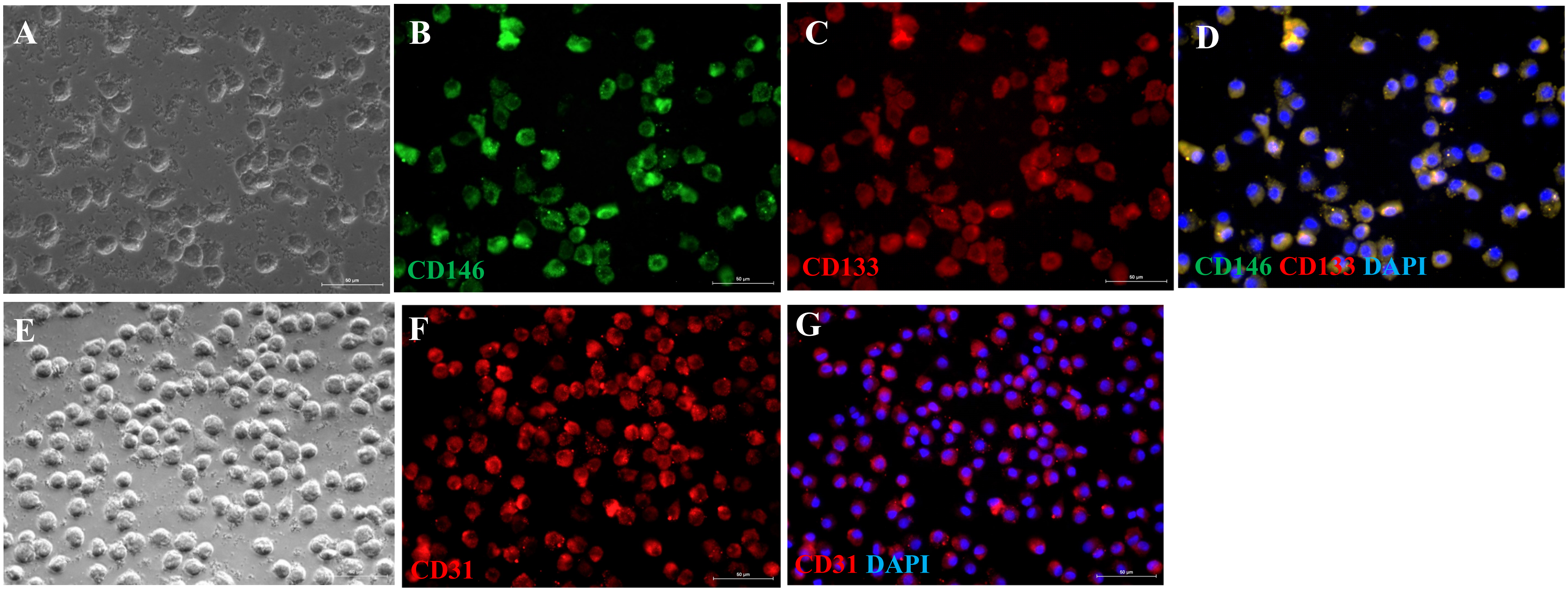

Supplementary Figure S1: BOEC characterization. (A, E) Phase contrast images showing the morphology of blood outgrowth endothelial cells (BOECs). (B) Immunofluorescence staining for the vascular endothelial marker CD146 (green). (C) Staining for the progenitor marker CD133 (red). (D) Merged image of CD133 (red) and CD146 (green) with DAPI (blue) for nuclear staining, illustrating the co-expression of these markers in BOECs. (F) Staining for the endothelial marker CD31 (red). (G) Merged image of CD31 (red) with DAPI (blue), highlighting the localization of CD31 in BOECs.
